# *Helicobacter pylori* Eradication in the Era of Antibiotic Resistance: From Universal Regimens to Precision-Guided Management

**DOI:** 10.3390/antibiotics15070673

**Published:** 2026-07-09

**Authors:** Ayman Elbehiry, Eman Marzouk

**Affiliations:** Department of Public Health, College of Applied Medical Sciences, Qassim University, P.O. Box 6666, Buraydah 51452, Saudi Arabia; e.marzouk@qu.edu.sa

**Keywords:** *Helicobacter pylori*, antibiotic resistance, susceptibility-guided therapy, molecular diagnostics, eradication therapy, precision-guided management, antimicrobial stewardship, resistance surveillance

## Abstract

*Helicobacter pylori* (*H. pylori*) infection represents a major global health challenge and a leading cause of peptic ulcer disease and gastric cancer. The increasing prevalence of antimicrobial resistance has progressively reduced the effectiveness of traditional empirical eradication regimens, highlighting the need for more individualized treatment approaches. This review evaluates the evolving transition from empirical therapy toward precision-guided *H. pylori* management by integrating current evidence on resistance epidemiology, determinants of eradication failure, advances in molecular diagnostics, and susceptibility-guided treatment strategies. Contemporary and emerging therapeutic approaches are evaluated in the context of changing resistance patterns, with emphasis on their clinical applicability and potential contributions to treatment optimization. The review also addresses the practical challenges that continue to limit implementation of personalized management, including restricted access to resistance testing, infrastructure limitations, economic constraints, and global disparities in healthcare resources. Furthermore, emerging developments in rapid diagnostics, resistance surveillance, and data-driven treatment selection are considered as components of future management frameworks. Overall, the evidence supports moving away from one-size-fits-all empirical therapy toward targeted treatment based on local resistance patterns and epidemiological trends. Expanding access to effective diagnostics and optimizing treatment selection will be essential for improving eradication outcomes and preserving the long-term effectiveness of available antimicrobial agents.

## 1. Introduction

*Helicobacter pylori* (*H. pylori*) infection remains a major global health challenge because of its established association with peptic ulcer disease, chronic gastritis, mucosa-associated lymphoid tissue lymphoma, and gastric adenocarcinoma [1]. Persistent infection also represents an important preventable risk factor for gastric cancer and contributes substantially to long-term gastrointestinal morbidity worldwide [2,3]. Although the prevalence of *H. pylori* infection has declined in several developed countries, it continues to affect a substantial proportion of the global population. The burden remains especially high in developing regions and socioeconomically disadvantaged communities. Geographic variability in prevalence remains closely linked to differences in sanitation, healthcare accessibility, living conditions, and antibiotic exposure patterns [2,4].

For many years, clarithromycin-based triple therapy was considered the standard first-line eradication regimen because of its simplicity, acceptable tolerability, and initially high eradication rates. The combination of a proton pump inhibitor with clarithromycin and amoxicillin or metronidazole achieved favorable eradication outcomes in many populations and became widely adopted in routine clinical practice [5,6]. However, the effectiveness of conventional empirical therapy has declined considerably during the past two decades. Increasing resistance to clarithromycin, metronidazole, and fluoroquinolones has reduced eradication success rates in many geographic regions and restricted the reliability of traditional empirical regimens. Recent systematic reviews and multicenter surveillance studies have shown that resistance to commonly prescribed antibiotics now exceeds acceptable treatment thresholds in many settings worldwide [7,8,9].

Increasing geographic variation in antimicrobial resistance (AMR) has further complicated treatment selection. Regimens that maintain efficacy in some regions may produce substantially lower eradication rates in areas characterized by elevated macrolide or fluoroquinolone resistance. Accordingly, universal treatment strategies are becoming increasingly difficult to justify without consideration of regional resistance epidemiology and prior antibiotic exposure [10,11,12]. At the same time, eradication failure is recognized as a multifactorial process that extends beyond AMR alone. Host-related factors, including poor adherence, inadequate acid suppression, treatment intolerance, aging-associated physiological changes, and previous antibiotic exposure, may significantly influence eradication outcomes. In addition, bacterial adaptive mechanisms such as biofilm formation, heteroresistance, and genetic diversity may contribute to persistent infection and reduced treatment response [8,13,14].

**Figure 1:**
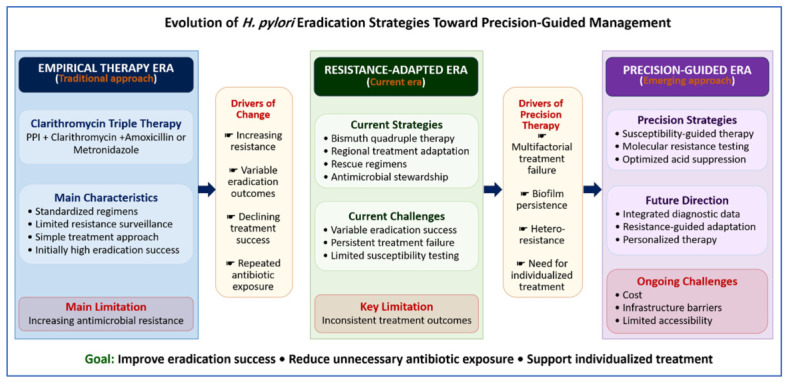
Evolution of *H. pylori* eradication strategies from empirical therapy toward precision-guided management. Increasing AMR, variable eradication outcomes, and persistent eradication failure have driven the transition from standardized treatment regimens to more individualized therapeutic approaches. Contemporary management incorporates resistance surveillance, susceptibility-guided therapy, and optimized treatment selection to improve eradication success while reducing unnecessary antibiotic exposure.

These therapeutic challenges have accelerated an important transition in the management of *H. pylori* infection. Current strategies are moving away from simplified empirical regimens toward more targeted and resistance-informed therapeutic approaches aimed at optimizing eradication success while reducing unnecessary antibiotic exposure (Figure 1). Recent international consensus reports emphasize the importance of antimicrobial stewardship, regional resistance surveillance, and susceptibility-guided therapy. These approaches are especially important in areas with high clarithromycin resistance or frequent treatment failure [6,11,12]. Advances in molecular diagnostics and resistance detection technologies have further strengthened the movement toward precision-guided eradication strategies. Polymerase chain reaction (PCR)-based resistance testing and emerging noninvasive molecular approaches are being investigated as tools for individualized therapeutic selection and optimized antibiotic stewardship [12,14,15].

Precision-guided management of *H. pylori* infection involves more than susceptibility-guided therapy alone. Its goal is to select the most appropriate treatment for each patient by combining antimicrobial susceptibility results with other clinically relevant information. These include previous antibiotic exposure, regional resistance patterns, treatment history, and patient-related factors that may influence treatment response. Recent international guidelines recommend individualizing eradication therapy whenever possible by integrating these factors into clinical decision-making [3,6,12]. Therefore, susceptibility-guided therapy should be regarded as one component of precision-guided management rather than its complete definition. Precision-guided management combines microbiological, clinical, and epidemiological information to tailor treatment. This includes susceptibility results, previous antibiotic use, regional resistance patterns, treatment history, and patient characteristics. This broader approach aims to improve eradication success while reducing unnecessary antibiotic use and supporting antimicrobial stewardship [3,12].

In parallel, treatment options for *H. pylori* infection continue to evolve. Bismuth quadruple therapy (BQT), vonoprazan-based regimens, high-dose dual therapy (HDDT), and optimized salvage therapies have become important alternatives to conventional triple therapy, especially in regions with increasing AMR [3,16]. However, precision-guided management is still difficult to implement in many healthcare systems because access to susceptibility testing remains limited. Economic constraints, laboratory infrastructure, and regional differences in healthcare resources also affect its routine use [12]. Treatment decisions should therefore consider the clinical setting, previous antibiotic exposure, regional resistance patterns, and the availability of susceptibility testing [3,12].

Accordingly, this review goes beyond summarizing the available evidence. It integrates current knowledge on AMR, determinants of eradication failure, diagnostic advances, and treatment strategies into a practical framework for precision-guided management. Based on the available evidence, we propose that successful *H. pylori* eradication depends not only on choosing the right antibiotics but also on considering patient characteristics, regional resistance patterns, and available healthcare resources when making treatment decisions. This perspective may improve eradication success while supporting antimicrobial stewardship.

## 2. Literature Search Strategy

This narrative review provides a comprehensive overview of current challenges and emerging strategies in *H. pylori* management, with particular emphasis on AMR, susceptibility-guided therapy, precision-guided management, and emerging therapeutic approaches. Relevant literature published between January 2000 and March 2026 was identified through searches of PubMed, Scopus, Web of Science, and Google Scholar. The search strategy used combinations of keywords, including “*H. pylori*”, AMR, eradication therapy, susceptibility testing, molecular diagnostics, precision medicine, vonoprazan, HDDT, antimicrobial stewardship, and artificial intelligence (AI). Eligible publications included systematic reviews, meta-analyses, international consensus reports, clinical guidelines, randomized controlled trials, observational studies, and other clinically relevant investigations.

Priority was given to recent high-quality evidence, while seminal studies were included when necessary to provide historical context. Publications lacking direct relevance to the objectives of the review, duplicate reports, conference abstracts without full-text availability, and non-English articles were excluded. Reference lists of eligible articles were also screened to identify additional relevant studies. The retrieved evidence was critically evaluated and synthesized to provide a balanced overview of current knowledge, implementation challenges, and future directions in *H. pylori* management. Greater emphasis was placed on high-level evidence, including international clinical guidelines, umbrella reviews, systematic reviews, meta-analyses, and randomized controlled trials. Observational studies and earlier investigations were included primarily to provide supporting evidence or historical context. Generative artificial intelligence (ChatGPT-5.5; OpenAI, San Francisco, CA, USA) was used only to assist with language editing, improvement of grammar, sentence clarity, and manuscript organization. It was not used to generate scientific content, interpret data, select references, or draw scientific conclusions. All literature selection, critical evaluation, interpretation, and final manuscript preparation were performed and verified by the authors.

## 3. Global Antibiotic Resistance Patterns and Regional Therapeutic Variability

### 3.1. Global Resistance Trends

AMR is now one of the main causes of failed *H. pylori* eradication worldwide. Resistance rates vary widely across regions and continue to change because of differences in antibiotic use, prescribing practices, healthcare systems, and antimicrobial stewardship. Large international surveillance studies confirmed substantial regional heterogeneity, particularly for clarithromycin, metronidazole, and fluoroquinolones [17,18].

Clarithromycin resistance is considered the most clinically relevant form of resistance because of its direct impact on standard triple therapy. Primary clarithromycin resistance exceeds the recommended threshold (above 15%) in many WHO regions [17]. Similar findings were confirmed by subsequent international analyses [19]. Therefore, empirical clarithromycin-based regimens have progressively lost therapeutic reliability in many populations.

Recent international guidelines discourage routine empirical clarithromycin-containing therapy when local susceptibility data are unavailable. They also recommend avoiding these regimens in regions with high clarithromycin resistance [20,21]. These findings establish clarithromycin resistance as a major global determinant of treatment failure and explain the declining effectiveness of empirical clarithromycin-based triple therapy [17,20,21].

Several molecular mechanisms contribute to clarithromycin resistance, particularly point mutations involving the 23S rRNA gene. Among these, the A2143G mutation is one of the most frequently identified resistance-associated variants [22]. Beyond specific resistance-associated mutations, *H. pylori* has several genetic adaptation mechanisms that promote survival under antimicrobial pressure and contribute to the development of AMR [23]. Metronidazole resistance is also highly prevalent worldwide, especially in developing regions where nitroimidazole exposure is common. Global surveillance studies demonstrated resistance rates exceeding 30% in several populations [17,24]. Although optimized bismuth-containing regimens may partially overcome reduced susceptibility, elevated prevalence continues to compromise eradication outcomes. Resistance mechanisms frequently involve alterations in reductase-associated genes, including *rdxA* and *frxA* [22].

Fluoroquinolone resistance has emerged as another major therapeutic concern, particularly because levofloxacin-containing regimens are widely used after first-line treatment failure. International surveillance studies demonstrated progressively higher resistance prevalence across Asia, Europe, and North America [17,18]. Mutations involving the *gyrA* gene are strongly associated with reduced fluoroquinolone susceptibility and lower eradication success [22]. Multidrug resistance is becoming more common. Simultaneous resistance to clarithromycin, metronidazole, and fluoroquinolones has been reported in multiple geographic regions, particularly in settings characterized by repeated antibiotic exposure and limited stewardship programs [22,25]. These multidrug-resistant strains substantially restrict empirical treatment options and complicate rescue therapy selection. The increasing prevalence of multidrug resistance also highlights the limitations of relying solely on empirical therapy. As resistance patterns become more complex, treatment selection increasingly depends on regional surveillance data and, whenever available, antimicrobial susceptibility testing (AST) to improve the likelihood of successful eradication [20,21].

Although AMR is a major determinant of treatment failure, conventional susceptibility profiles do not fully explain persistent infection. Emerging evidence suggests that adaptive bacterial mechanisms, including biofilm formation, heteroresistance, and phenotypic tolerance, may contribute to reduced treatment response and survival during antibiotic exposure. These factors are discussed in greater detail in Section 3.3.

### 3.2. Geographic Differences in Eradication Success

Geographic heterogeneity in AMR directly influences the effectiveness of *H. pylori* eradication regimens. Therapeutic strategies that achieve acceptable outcomes in one population may produce substantially lower success rates in regions characterized by different resistance profiles [17,26]. Marked regional differences have been reported across Asia, Europe, North America, the Middle East, and Africa. Several Asian countries demonstrated elevated clarithromycin and fluoroquinolone resistance prevalence, whereas metronidazole resistance remains particularly high in many developing regions [17,24]. In several Middle Eastern populations, clarithromycin resistance has reached levels that substantially reduce the efficacy of standard triple therapy [25]. Regional antibiotic exposure patterns strongly influence resistance epidemiology. European surveillance studies demonstrated significant associations between community macrolide use and clarithromycin resistance prevalence [17]. Similar relationships have been observed between fluoroquinolone exposure and declining efficacy of levofloxacin-containing rescue regimens [18]. These observations explain why eradication regimens successful in one region may fail in another despite similar guideline recommendations.

As resistance prevalence increased globally, therapeutic recommendations progressively shifted away from universal empirical therapy toward resistance-informed treatment adaptation. Current international guidelines recommend BQT, susceptibility-guided treatment, or optimized alternative regimens in regions characterized by elevated clarithromycin resistance [20,21]. Regional variability also contributes to differences in eradication outcomes reported across clinical trials and meta-analyses. Resistance prevalence can significantly influence the efficacy of empirical eradication regimens, particularly clarithromycin-containing therapies [26].

Thus, successful eradication depends on integrating local resistance epidemiology into therapeutic decision-making rather than relying on standardized treatment regimens. Overall, the available evidence indicates that treatment recommendations should remain flexible rather than universal. Continuous regional surveillance is therefore essential to ensure that empirical regimens remain aligned with current resistance patterns and local epidemiology.

### 3.3. Limitations of Current Resistance Surveillance

Despite increasing awareness of AMR in *H. pylori* infection, important limitations continue to affect current surveillance systems worldwide. Resistance data remain incomplete in many low- and middle-income countries where access to culture-based susceptibility testing and molecular diagnostics is restricted [18,27]. Another major challenge is the substantial heterogeneity among published studies. Differences in diagnostic methods, susceptibility testing techniques, study populations, and regional sampling strategies complicate direct comparison of resistance prevalence across healthcare systems [17].

Several geographic regions also remain underrepresented in global surveillance datasets, predominantly parts of Africa and other resource-limited healthcare settings [27]. This underrepresentation can delay regional adaptation of treatment recommendations and contribute to continued use of suboptimal empirical regimens. Resistance prevalence additionally changes over time. Longitudinal surveillance studies from Asia, Europe, and North America demonstrated progressively higher clarithromycin and fluoroquinolone resistance during recent decades [17,18]. Consequently, empirical regimens that were previously effective may rapidly lose therapeutic reliability if surveillance systems are not continuously updated.

Real-world implementation barriers further complicate surveillance-guided management. Several international studies reported limited availability of AST, restricted access to molecular diagnostics, reimbursement limitations, and inconsistent incorporation of resistance data into routine clinical practice [18,28]. These limitations highlight the need for expanded surveillance networks, broader access to molecular resistance testing, and stronger antimicrobial stewardship programs. Improved real-time resistance monitoring will likely become essential for supporting resistance-informed *H. pylori* management.

Despite these limitations, current surveillance data consistently demonstrate that AMR has become a major driver of treatment failure worldwide. Expanding surveillance programs and improving access to molecular diagnostics should therefore be considered public health priorities. Improved surveillance will support more informed treatment decisions, facilitate broader implementation of precision-guided management, and help preserve the effectiveness of currently available eradication regimens. The major regional patterns of AMR and their corresponding therapeutic implications are summarized in Table 1.

Overall, the evidence presented in this section demonstrates that AMR is now a central determinant of *H. pylori* treatment success. Marked geographic variation and the continued evolution of resistance patterns limit the effectiveness of universal empirical regimens. These findings support the growing need for treatment strategies that integrate regional epidemiology, surveillance data, and AST whenever feasible. The following section discusses additional determinants of eradication failure beyond AMR that further influence eradication outcomes.

## 4. Determinants of Eradication Failure Beyond Antibiotic Resistance

### 4.1. Host-Related Determinants

Successful *H. pylori* eradication depends not only on antimicrobial susceptibility but also on several host-related factors that influence treatment effectiveness. Among these, adherence to therapy, treatment tolerability, and variability in acid suppression play particularly important roles [29,30]. Poor adherence is one of the strongest and most consistent predictors of eradication failure. Multidrug regimens, prolonged treatment courses, adverse events, and inadequate patient education can reduce treatment adherence [31,32]. Patients who do not complete therapy consistently achieve lower eradication rates than those with good adherence [6,32,33]. In a pediatric cohort, eradication approached 90% among patients receiving more than 90% of prescribed medications but declined substantially in non-adherent individuals [33]. Similar findings have been reported in large real-world studies, where compliance was the factor most strongly associated with successful treatment [32].

Treatment intolerance may further compromise outcomes. Although most adverse events are mild, symptoms such as nausea, diarrhea, abdominal discomfort, dysgeusia, and vomiting can discourage patients from completing therapy [32,34]. Adverse events have repeatedly been associated with lower compliance, whereas educational support and structured follow-up improve adherence and treatment success [32,35,36]. Effective acid suppression is another prerequisite for successful eradication. Higher intragastric pH improves antibiotic stability and enhances bacterial susceptibility to treatment [37,38]. However, substantial interindividual variability exists in acid control, which may contribute to different eradication outcomes despite the use of identical regimens [37,38].

Genetic variation in cytochrome P450 2C19 (CYP2C19), a key enzyme involved in proton pump inhibitor metabolism, is an important source of this variability. Rapid metabolizers may achieve lower acid suppression than poor metabolizers, resulting in reduced treatment efficacy [37]. Several studies have reported higher eradication rates among poor metabolizers, particularly in regimens that rely heavily on proton pump inhibitor activity [37,38]. Overall, the available evidence consistently identifies poor adherence, inadequate acid suppression, and treatment intolerance as the most important host-related predictors of eradication failure. Unlike AMR, many of these factors are potentially modifiable through patient education, optimized acid suppression, and careful treatment selection. Addressing these modifiable determinants may substantially improve eradication success without changing the antimicrobial regimen itself [6,29,32,38]. Lifestyle and metabolic characteristics may also influence treatment response. These factors are discussed separately because they represent potentially modifiable determinants of eradication success.

### 4.2. Lifestyle and Metabolic Factors

Lifestyle and metabolic factors may also contribute to variability in *H. pylori* eradication outcomes. Although their influence is less direct than AMR, increasing evidence suggests that obesity, metabolic dysfunction, and physical inactivity can affect treatment response [39,40]. Obesity has emerged as a clinically relevant predictor of eradication failure. In a prospective study, eradication was achieved in 55.0% of overweight or obese patients compared with 85.4% of individuals with normal body weight, and body mass index was identified as an independent predictor of treatment failure [41]. Other studies have suggested that obesity-associated alterations in drug distribution and pharmacokinetics may contribute to reduced treatment effectiveness [39,41].

Metabolic dysfunction may further influence outcomes. Associations have been reported between *H. pylori* infection and metabolic syndrome, insulin resistance, dyslipidemia, and fatty liver disease [40,42]. Obesity-related inflammation and immune dysregulation may create conditions that are less favorable for successful eradication [40,43]. Physical inactivity is closely linked to obesity and metabolic impairment. Conversely, regular physical activity is associated with improved metabolic health and reduced systemic inflammation [43,44]. Current evidence consistently identifies obesity as the lifestyle factor most strongly associated with reduced eradication success. By contrast, the independent contributions of metabolic dysfunction and physical inactivity remain uncertain. Well-designed prospective studies are needed to determine whether modifying these factors can directly improve eradication success [39,40,41,43,45].

### 4.3. Bacterial Adaptive Mechanisms

Several bacterial adaptive mechanisms may contribute to eradication failure independently of conventional AMR. These mechanisms enable *H. pylori* to persist within the gastric environment despite apparently appropriate therapy [8,46,47]. Biofilm formation is one of the most important persistence strategies. Within biofilms, bacterial cells are enclosed in an extracellular matrix that reduces antibiotic penetration and promotes survival under antimicrobial stress [8,46]. Biofilm-associated organisms may therefore be more difficult to eradicate than planktonic bacteria despite similar susceptibility profiles [8].

Heteroresistance presents an additional challenge. The coexistence of susceptible and resistant bacterial subpopulations within the same host may lead to incomplete eradication when treatment eliminates only part of the infecting population [8]. This phenomenon may also reduce the predictive value of routine susceptibility testing. *H. pylori* may also persist within gastric epithelial cells. Intracellular localization can provide temporary protection from both antimicrobial agents and host immune responses, potentially allowing bacterial survival after treatment [48,49].

Bacterial burden represents another clinically relevant factor. Several studies have demonstrated lower eradication rates among patients with dense gastric colonization than among those with lower bacterial loads [50,51,52]. A larger bacterial population may require greater antimicrobial exposure to achieve complete clearance and may increase the likelihood of persistent infection. Collectively, these adaptive mechanisms help explain why treatment failure may occur even when conventional susceptibility testing predicts antibiotic sensitivity. Their recognition highlights the limitations of relying solely on resistance testing and supports the development of more comprehensive approaches to individualized treatment selection [8,46,47].

### 4.4. Treatment-Related Limitations

Treatment failure may also result from limitations in treatment delivery rather than AMR alone. Factors such as inadequate treatment duration, insufficient drug exposure, and suboptimal regimen design can reduce eradication success [53,54,55]. Treatment duration is a major determinant of outcome. Large clinical studies and registry analyses have consistently shown that longer treatment courses achieve higher eradication rates than shorter regimens and are more likely to reach accepted therapeutic targets [53,54].

Suboptimal dosing may also compromise bacterial clearance. Insufficient antibiotic exposure can reduce treatment efficacy and increase the probability of persistent infection. Optimization studies have demonstrated that appropriately intensified dosing strategies can improve eradication success, particularly in difficult-to-treat populations [53]. Regimen complexity remains another practical limitation. Contemporary therapies frequently involve multiple medications administered over prolonged periods, increasing treatment burden and reducing the likelihood of full adherence [54,56]. Therapeutic success depends on selecting an appropriate regimen. It also requires patients to complete treatment as prescribed.

**Figure 2:**
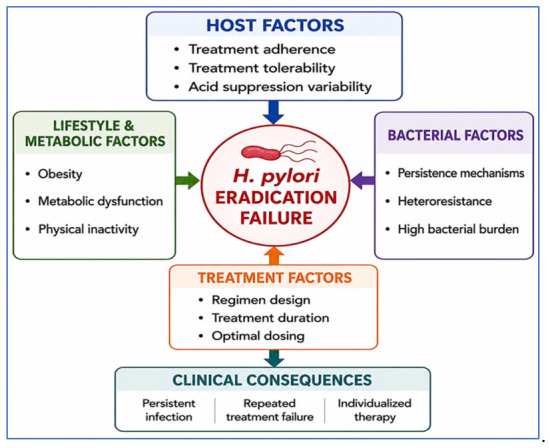
Multifactorial determinants contributing to *H. pylori* eradication failure. Host-, lifestyle-, bacterial-, and treatment-related factors interact to influence eradication outcomes. Recognition of these interconnected determinants supports precision-guided management and individualized treatment strategies.

Importantly, optimization of treatment duration, dosing strategies, and regimen design can partially improve outcomes even in challenging clinical settings [53,54,55]. These observations highlight that treatment optimization should complement resistance-guided therapy rather than replace it. Unlike bacterial resistance, treatment-related limitations are largely preventable. Optimizing treatment duration, simplifying regimens when appropriate, improving patient counseling, and ensuring adequate drug exposure are practical strategies that can improve eradication success without requiring new antimicrobial agents [53,54,55,56]. The major host-, bacterial-, and treatment-related determinants of *H. pylori* eradication failure are summarized in Table 2, while their interactions and clinical implications are illustrated in Figure 2.

Taken together, the evidence demonstrates that eradication failure is a multifactorial process rather than a consequence of AMR alone. Host characteristics, bacterial adaptive mechanisms, and treatment-related factors frequently interact to reduce therapeutic success. Recognizing these determinants provides the foundation for precision-guided management, in which treatment decisions should integrate patient characteristics, bacterial factors, and treatment-related considerations to maximize eradication success.

## 5. Transition Toward Precision-Guided *H. pylori* Management

Precision-guided management involves more than choosing antibiotics according to susceptibility testing results. Treatment decisions should also consider previous eradication history, prior antibiotic exposure, regional resistance patterns, patient characteristics, and the resources available within each healthcare system [3,12]. Within this framework, susceptibility-guided therapy is one important tool for selecting individualized treatment. The overall goal is to improve eradication success while avoiding unnecessary antibiotic exposure and supporting antimicrobial stewardship [3,6,12].

### 5.1. Limitations of Universal Empirical Therapy

For many years, empirical therapy has been the standard approach for *H. pylori* eradication because treatment can be initiated without prior susceptibility testing. However, increasing AMR has progressively reduced the reliability of this strategy. As resistance patterns vary substantially among regions, eradication outcomes have become progressively dependent on local epidemiology rather than on universal treatment recommendations [54,57]. Data from the European Registry on *H. pylori* Management (Hp-EuReg) demonstrated substantial variability in treatment practices and eradication outcomes across participating countries, emphasizing the limitations of a universal therapeutic strategy [54]. Consequently, international guidelines recommend adapting treatment selection according to regional resistance epidemiology and, when available, individual susceptibility data [3,20,58].

Despite these limitations, empirical therapy remains the most widely used approach in routine clinical practice because susceptibility testing is not universally available. Differences in healthcare infrastructure, laboratory capacity, cost, and access to diagnostic testing continue to influence treatment selection worldwide [3,59]. Consequently, growing therapeutic variability has accelerated interest in individualized approaches that incorporate resistance information into treatment decision-making.

The choice between empirical and resistance-guided therapy should be determined by the clinical setting. Empirical treatment remains appropriate where local resistance patterns support its use or where susceptibility testing is not readily available. However, resistance-guided treatment is preferable in regions with high clarithromycin resistance, after previous eradication failure, or whenever reliable susceptibility data can be obtained. Selecting therapy according to these factors increases the likelihood of successful eradication while reducing unnecessary antibiotic exposure and supporting antimicrobial stewardship [3,20,58].

### 5.2. Phenotypic Susceptibility Testing

Phenotypic susceptibility testing remains the reference approach for assessing AMR in *H. pylori* because it directly measures bacterial growth in the presence of antibiotics. Unlike molecular methods, which infer resistance from specific genetic alterations, phenotypic testing evaluates the overall susceptibility profile of a cultured isolate and provides information across multiple antimicrobial classes. Mégraud and Lehours emphasized that culture-based testing continues to serve as the foundation for resistance characterization and antimicrobial surveillance [60]. Successful phenotypic testing requires isolation of viable *H. pylori* from gastric biopsy specimens obtained during endoscopy. However, culture is technically demanding because the organism grows slowly, requires specialized media, and must be maintained under microaerophilic conditions. Diagnostic performance may also be influenced by specimen handling, transport conditions, and recent exposure to antibiotics or acid-suppressive therapy. These factors have limited the routine implementation of culture-based testing in many clinical settings [60,61].

Among available methods, agar dilution remains the reference standard for determining minimum inhibitory concentrations (MICs). The technique provides highly reproducible quantitative results and serves as the benchmark against which alternative susceptibility methods are evaluated. Nevertheless, agar dilution is labor-intensive, requires preparation of multiple antibiotic-containing media, and is generally restricted to specialized laboratories [60,62]. The E-test was developed as a simpler alternative that generates quantitative MIC values using antibiotic gradient strips. Several studies have demonstrated good agreement between E-test and agar dilution results for clinically important antibiotics, supporting its use in routine susceptibility testing when standardized protocols are applied [62,63,64].

In clinical practice, phenotypic susceptibility testing is particularly useful for patients with previous eradication failure, multiple unsuccessful treatment attempts, or suspected multidrug-resistant infection. When culture facilities and technical expertise are available, these methods provide comprehensive susceptibility profiles that support selection of effective salvage therapy and contribute to regional AMR surveillance [3,60,61].

Despite their diagnostic value, phenotypic methods face important practical limitations. Culture failure may occur because of low bacterial density, uneven gastric colonization, or suboptimal specimen handling. In addition, prolonged turnaround times, specialized laboratory expertise, and limited availability continue to restrict access to susceptibility testing in many regions. Ng et al. highlighted that these barriers remain major obstacles to the widespread implementation of culture-guided management, particularly in resource-limited settings [61]. Phenotypic susceptibility testing provides direct and clinically relevant resistance information. However, its complexity and limited availability restrict routine clinical use. These limitations have increased interest in molecular approaches that provide resistance results more rapidly and require fewer laboratory resources.

### 5.3. Molecular Resistance Detection

The limitations of culture-based susceptibility testing have accelerated the development of molecular methods for resistance detection in *H. pylori*. Unlike phenotypic approaches, molecular assays identify resistance-associated genetic alterations directly from bacterial isolates or clinical specimens without requiring bacterial culture. This capability shortens turnaround time and improves access to resistance testing in routine clinical practice [65,66]. Detection of clarithromycin resistance represents the most established clinical application of molecular testing. Point mutations within the 23S rRNA gene, particularly A2142G and A2143G substitutions, are strongly associated with treatment failure and can be identified directly from gastric biopsy specimens. Cambau et al. demonstrated high diagnostic accuracy for molecular detection of clarithromycin-resistant strains, and these mutations remain the principal markers used to guide clarithromycin-containing therapy [67].

Molecular approaches have also improved the detection of fluoroquinolone resistance. Mutations involving codons 87 and 91 of the *gyrA* gene are closely associated with reduced susceptibility to levofloxacin and can be identified using PCR-based assays, sequencing platforms, and commercial hybridization systems. Multiple studies have demonstrated strong concordance between these mutations and phenotypic resistance, supporting their clinical utility in treatment selection [67,68]. Several molecular platforms are currently available, including real-time PCR, line probe assays, targeted sequencing, and whole-genome sequencing. PCR-based methods remain the most widely used because they are rapid, relatively inexpensive, and suitable for routine diagnostic laboratories. Sequencing technologies provide broader genetic information and enable simultaneous analysis of multiple resistance-associated loci, facilitating detection of emerging resistance determinants [69,70].

Despite these advantages, molecular resistance testing has important limitations. Not all resistance mechanisms are explained by well-characterized genetic markers. Prediction of metronidazole resistance remains challenging because multiple genetic pathways may contribute to the resistant phenotype. In addition, the presence of a resistance-associated mutation does not always correspond to complete phenotypic resistance, whereas resistant isolates may occasionally lack recognized genetic markers. Consequently, genotype–phenotype concordance remains strongest for clarithromycin and fluoroquinolone resistance but less reliable for several other antibiotics [68,69]. Molecular assays can also detect mixed bacterial populations containing both susceptible and resistant subpopulations within the same host. Cambau et al. reported frequent identification of mixed genotypes, reflecting either coinfection with multiple strains or coexistence of susceptible and resistant variants [67].

Molecular testing has become increasingly valuable in routine clinical practice because it provides clinically relevant resistance information within a short timeframe. It is particularly useful when rapid treatment decisions are required or when culture-based testing is unavailable. In appropriately resourced healthcare settings, molecular diagnostics can support both first-line treatment selection and management of patients who require rescue therapy [65,66,67,68,69].

Molecular resistance testing provides rapid identification of clinically relevant resistance markers and has become an important tool for treatment selection, particularly when culture-based susceptibility testing is unavailable or impractical. Although it does not completely replace phenotypic susceptibility testing, it has become an important component of contemporary precision-guided management strategies.

### 5.4. Clinical Applications of Tailored Therapy

The principal objective of susceptibility testing is to support selection of the most appropriate eradication regimen for an individual patient. By identifying resistance-associated characteristics before treatment, tailored therapy can reduce exposure to ineffective antibiotics and improve the likelihood of successful eradication [71]. The clinical value of individualized treatment is particularly evident during first-line therapy selection. Molecular detection of clarithromycin resistance allows susceptible infections to be treated with clarithromycin-containing regimens while directing resistant cases toward alternative strategies. Tailored therapy achieved higher eradication rates and reduced overall medical costs than empirical treatment [72].

Resistance-guided treatment may provide even greater benefit after eradication failure. Patients requiring salvage therapy frequently harbor strains with acquired resistance to previously administered antibiotics, reducing the effectiveness of empirical retreatment. Tailored bismuth-based quadruple therapy achieved higher eradication rates than empirical levofloxacin-based rescue therapy after failure of clarithromycin-containing regimens [73]. Similar observations were reported by Liou et al., who demonstrated superior outcomes with genotypic resistance-guided treatment in refractory infection [74]. Recent evidence suggests that molecular testing may provide a practical alternative to conventional culture-guided management. Chen et al. reported eradication outcomes with molecular testing-guided therapy that were comparable to those achieved using culture-based susceptibility testing, supporting the integration of molecular diagnostics into routine clinical practice where culture facilities are limited [75].

Randomized studies have generally shown that susceptibility-guided therapy improves eradication rates by about 5–15% compared with empirical treatment. However, the size of this benefit varies according to local resistance patterns, previous antibiotic exposure, and patient selection [72,73,74,75].

Beyond improving eradication success, individualized treatment may support antimicrobial stewardship by reducing unnecessary exposure to ineffective antibiotics. For this reason, current consensus reports advocate greater incorporation of resistance testing into treatment algorithms whenever feasible [3]. Despite these advantages, implementation remains uneven across healthcare systems. Access to culture facilities, molecular diagnostic platforms, trained personnel, and reimbursement mechanisms varies substantially among regions. In many settings, susceptibility testing is not routinely available before initial treatment, limiting the practical application of individualized management strategies. No single diagnostic or therapeutic strategy is appropriate for every patient or healthcare setting. The optimal approach should be guided by regional resistance patterns, previous antibiotic exposure, patient characteristics, availability of diagnostic testing, and local healthcare resources. This flexible approach allows clinicians to balance scientific evidence with practical considerations when selecting eradication therapy [3,12,58].

Importantly, therapeutic strategies should always be selected according to the clinical setting. Empirical first-line therapy remains appropriate where local resistance patterns support its use or susceptibility testing is unavailable. In contrast, susceptibility-guided treatment is particularly valuable after previous eradication failure, in patients with repeated antibiotic exposure, or in regions with high clarithromycin resistance where reliable susceptibility data can guide treatment selection. Treatment decisions should also consider patient-specific factors, including medication allergy, treatment tolerability, adherence, and prior antimicrobial use. Population-based *H. pylori* screening programs generally rely on locally recommended empirical regimens because individualized susceptibility testing is often not practical on a large scale [3,12,20,58,59].

Overall, the available evidence supports susceptibility-guided therapy as the preferred strategy whenever reliable resistance testing is available. However, successful precision-guided management requires more than susceptibility testing alone. Treatment decisions should also consider previous eradication history, regional resistance patterns, patient characteristics, treatment availability, and healthcare resources. Where susceptibility testing is unavailable, optimized empirical therapy based on current regional surveillance remains an appropriate and evidence-based alternative [3,12,58].

**Figure 3:**
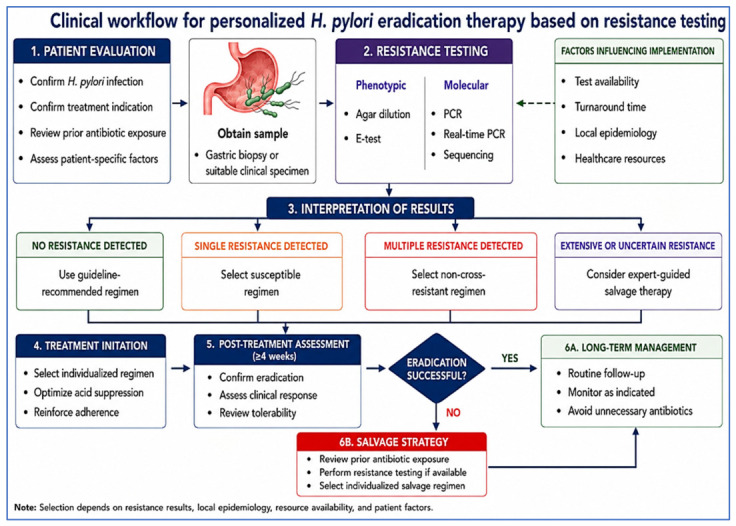
Precision-guided management pathway for *H. pylori* eradication. Resistance-testing results are integrated with previous treatment history, patient characteristics, and regional resistance patterns to support individualized treatment selection, post-treatment assessment, and salvage therapy.

The selection of the most appropriate resistance-testing method should be based on the clinical question, available laboratory resources, turnaround time, and local expertise. The principal characteristics, advantages, and limitations of phenotypic and molecular resistance-testing approaches are summarized in Table 3. The clinical workflow of personalized eradication therapy is illustrated in Figure 3. Once individualized treatment has been selected, the next challenge is choosing the most appropriate eradication regimen. The following section reviews current and emerging therapeutic strategies within this precision-guided framework.

## 6. Contemporary and Emerging Eradication Strategies

### 6.1. Optimized First-Line Eradication Strategies

The progressive decline in the effectiveness of conventional clarithromycin-based triple therapy has accelerated the adoption of alternative first-line regimens capable of maintaining high eradication rates despite increasing AMR. Recent treatment strategies seek to improve eradication outcomes through optimization of antibiotic selection, acid suppression, and treatment duration while minimizing unnecessary antibiotic exposure and preserving treatment tolerability [76,77,78].

Among currently available options, BQT remains one of the most reliable empirical first-line regimens, particularly in regions with moderate-to-high resistance rates. The therapeutic contribution of bismuth extends beyond direct antibacterial activity, as it enhances antibiotic efficacy and may partially mitigate the impact of resistance to key antimicrobial agents [79]. Multiple randomized trials and meta-analyses have consistently demonstrated that BQT achieves high and reproducible eradication rates across diverse populations while maintaining effectiveness in settings where clarithromycin-containing regimens perform less favorably [80,81,82]. Evidence also indicates that treatment optimization remains important even within this regimen class. Longer treatment durations, particularly 10–14-day courses, are generally associated with superior eradication outcomes compared with shorter regimens, with tetracycline-containing combinations showing particularly favorable results [83,84]. Accordingly, BQT has become a cornerstone empirical strategy in many present treatment settings. Across recent randomized trials and meta-analyses, optimized BQT has consistently achieved eradication rates above 90%, including in many regions where clarithromycin resistance is common [80,81,82,83,84].

Advances in acid suppression have further expanded first-line therapeutic options. Vonoprazan, a potassium-competitive acid blocker, provides more rapid, potent, and sustained gastric acid suppression than conventional proton pump inhibitors, thereby creating conditions that enhance antibiotic activity against *H. pylori* [85]. Accumulating evidence indicates that vonoprazan-based regimens achieve eradication rates that are at least comparable and often superior to those obtained with traditional PPI-based therapies, without compromising safety or tolerability [86,87,88]. The clinical advantage of vonoprazan appears particularly relevant in infections involving clarithromycin-resistant strains, where enhanced acid control may help improve treatment efficacy [89]. Recent studies have also reported eradication rates above 90% with several vonoprazan-based regimens, supporting their role as effective first-line treatment options [86,87,88,89,90,91]. More recently, simplified vonoprazan-amoxicillin dual therapy has attracted considerable interest as a strategy that reduces regimen complexity and limits exposure to multiple antibiotics while maintaining favorable eradication outcomes. Randomized trials and subsequent pooled analyses suggest that optimized dual therapy can achieve efficacy comparable to established quadruple regimens in appropriately selected patients [90,91].

A similar effort to simplify treatment while preserving effectiveness has driven interest in HDDT. This approach combines intensive acid suppression with frequent administration of high-dose amoxicillin to maintain sustained intragastric antibiotic exposure. Clinical studies have demonstrated eradication rates exceeding 90% in several treatment settings, with efficacy comparable to more complex multidrug regimens [92,93,94,95]. Furthermore, HDDT offers potential advantages in reducing overall antibiotic burden, limiting exposure to agents affected by widespread resistance, and improving treatment adherence through a more focused therapeutic strategy [96,97]. Nevertheless, successful implementation depends on strict dosing schedules and adequate acid suppression, factors that may influence its applicability in routine clinical practice.

These regimens provide complementary first-line options that can be selected according to regional resistance patterns, patient characteristics, and healthcare resources. BQT, vonoprazan-based regimens, and HDDT represent effective first-line options whose relative performance depends on regional resistance patterns, treatment availability, and patient-specific factors. Current evidence supports selecting therapy according to local epidemiology and clinical context rather than relying on a universally preferred regimen [88,98].

Overall, no single first-line regimen is universally superior across all clinical settings. The optimal choice depends on regional resistance patterns, previous antibiotic exposure, drug availability, patient characteristics, and local guideline recommendations. This evidence supports individualized regimen selection rather than a uniform treatment approach [3,58,88].

### 6.2. Salvage and Rescue Treatment Strategies

Despite substantial advances in first-line eradication therapy, treatment failure remains a clinically important challenge. Patients who do not achieve successful eradication require carefully selected retreatment strategies because repeated antibiotic exposure progressively limits therapeutic options and increases the likelihood of resistance. Thus, the selection of salvage therapy should consider previous treatment history, prior antibiotic exposure, local resistance patterns, and, whenever available, susceptibility testing results [99,100]. Levofloxacin-based regimens emerged as attractive salvage options because of their relative simplicity, favorable tolerability, and initially high eradication rates. Early clinical studies demonstrated that levofloxacin-containing triple therapy achieved eradication rates comparable or superior to traditional bismuth-based rescue regimens while being associated with fewer adverse effects and improved treatment adherence [99,101,102]. Subsequent investigations confirmed the efficacy of levofloxacin-containing therapies as second-line and third-line treatment options, supporting their incorporation into rescue treatment algorithms after failure of clarithromycin-based regimens [103,104,105].

However, the long-term effectiveness of levofloxacin-based therapy has been challenged by the global rise in fluoroquinolone resistance. Several studies demonstrated that eradication success declines substantially in the presence of levofloxacin-resistant strains, largely due to mutations affecting bacterial DNA gyrase. Therefore, eradication outcomes have become progressively less predictable in regions with high fluoroquinolone resistance rates, limiting the empirical use of levofloxacin-containing regimens [106,107]. Real-world observational data further indicate that although levofloxacin-based rescue therapy remains effective in selected settings, resistance trends must be carefully considered when choosing this approach [108].

For patients with multiple eradication failures, rifabutin-based therapy has emerged as an important rescue option. The clinical rationale for rifabutin use is supported by the relatively low prevalence of resistance compared with other commonly used antibiotics. Studies evaluating rifabutin-containing regimens have reported eradication rates of approximately 80–90% in patients with multiple previous treatment failures, although results vary between clinical settings [109,110,111,112,113]. More recently, the development of optimized rifabutin-based combinations has renewed interest in this strategy. In a large randomized trial, rifabutin-based therapy achieved significantly higher eradication rates than comparator treatment while maintaining an acceptable safety profile [111].

Nonetheless, rifabutin therapy is not without limitations. Because rifabutin is also used for the treatment of mycobacterial infections, its widespread use raises concerns regarding antimicrobial stewardship and the potential development of resistance in non-*H. pylori* pathogens. In addition, clinicians should remain aware of uncommon but potentially important adverse effects, including hematologic toxicity, particularly when prolonged treatment courses are considered [112]. For these reasons, most experts reserve rifabutin-based therapy for carefully selected patients with refractory infection after failure of standard rescue regimens [112,113].

Recent studies suggest that salvage therapy should be individualized according to prior treatment exposure and resistance patterns. Successful retreatment depends on therapeutic selection guided by previous antibiotic exposure, regional resistance epidemiology, treatment availability, and patient-specific clinical factors. As the number of prior eradication failures increases, the value of susceptibility-guided treatment becomes progressively greater, helping clinicians avoid ineffective antibiotic reuse and improve the likelihood of successful eradication [100,114].

Accordingly, modern rescue strategies are evolving from empiric retreatment toward a more personalized approach that seeks to maximize eradication success while preserving future treatment options. Whenever feasible, susceptibility testing should be considered before selecting rescue therapy, particularly after repeated eradication failures, to reduce unnecessary antibiotic reuse and improve the probability of successful treatment [3,100,114].

### 6.3. Adjunctive Therapeutic Optimization: Probiotics and Treatment Adherence

Despite advances in antimicrobial regimens, eradication failure remains influenced not only by bacterial resistance but also by treatment-related adverse events and suboptimal adherence. Consequently, adjunctive strategies that improve treatment tolerability and support completion of therapy have attracted increasing attention. Among these, probiotics have been the most extensively investigated. Experimental and clinical studies suggest that selected probiotic strains may interfere with *H. pylori* colonization, modulate local inflammatory responses, enhance mucosal barrier function, and help restore gastrointestinal microbial balance disrupted by antibiotic exposure. However, probiotics should be regarded as supportive interventions rather than independent eradication therapies and should always be used in combination with standard antimicrobial treatment [115,116].

Evidence from randomized controlled trials, meta-analyses, and umbrella reviews consistently indicates that adjunctive probiotic supplementation provides modest but reproducible clinical benefits. Overall, probiotics increase eradication rates by about 5–10% and consistently reduce treatment-related gastrointestinal adverse events, particularly diarrhea, nausea, and abdominal discomfort [117,118,119,120,121,122]. Better treatment tolerability may also improve adherence and contribute to successful eradication.

Despite these encouraging findings, important limitations remain. Considerable heterogeneity exists among studies regarding probiotic strains, formulations, doses, treatment duration, and timing of administration. Consequently, no single probiotic preparation can currently be recommended as the preferred adjunctive therapy for all patients [119,120,121,122].

Growing evidence also suggests that probiotic effects may be strain dependent. Lactobacillus-containing preparations have been among the most extensively studied and have shown favorable effects on both eradication rates and treatment tolerability [123]. Multi-strain formulations have also demonstrated potential benefits, particularly in reducing antibiotic-associated diarrhea and improving overall treatment tolerability [124]. Nevertheless, additional well-designed comparative studies are needed to identify the probiotic formulations that provide the greatest clinical benefit in different treatment settings.

Beyond probiotics, optimization of adherence remains a critical component of successful eradication therapy. Complex multidrug regimens, frequent dosing schedules, and treatment-related adverse events remain major barriers to treatment completion. Shrestha et al. demonstrated that inadequate adherence remains an important contributor to treatment failure, emphasizing the importance of patient education, simplified treatment strategies when feasible, and proactive management of adverse events [125]. These measures are inexpensive, widely applicable, and can substantially improve treatment success in routine clinical practice.

Overall, current evidence supports probiotics as adjunctive rather than primary therapy for *H. pylori* eradication. Their principal benefit lies in improving treatment tolerability and supporting adherence, whereas improvements in eradication rates are generally modest and may vary according to the probiotic strain and treatment regimen. Therefore, probiotic supplementation may be particularly useful for patients at increased risk of treatment-related adverse events or poor adherence rather than as a routine intervention for all patients [117,118,119,120,121,122,123,124].

**Figure 4:**
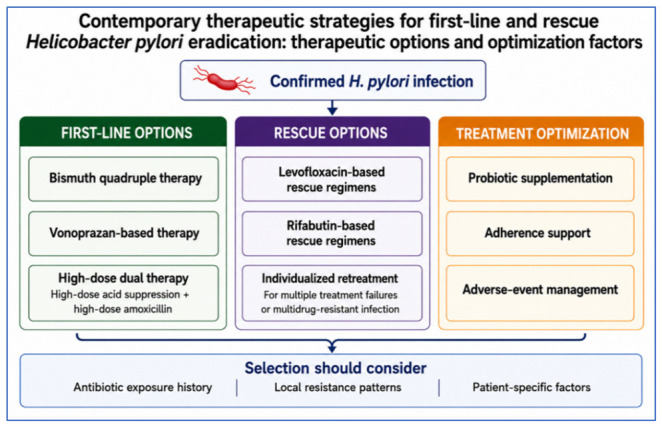
Recent therapeutic framework for the selection and optimization of first-line and rescue *H. pylori* eradication strategies. The figure summarizes the major therapeutic approaches currently used for *H. pylori* eradication, including first-line regimens, rescue treatment options following eradication failure, and adjunctive measures that may enhance treatment effectiveness. Therapeutic selection should integrate previous treatment history, regional resistance patterns, patient-related factors, and, whenever available, susceptibility testing to support precision-guided management.

A comparative summary of the principal current and emerging eradication regimens, including their therapeutic rationale, major advantages, limitations, and potential clinical applications, is presented in Table 4. To facilitate integration of these therapeutic options into clinical decision-making, Figure 4 illustrates a simplified framework for selecting and optimizing first-line and rescue eradication strategies according to treatment history and patient-specific considerations. Although these therapeutic strategies have substantially improved eradication outcomes, their successful implementation depends on more than clinical efficacy alone. Access to susceptibility testing, healthcare infrastructure, economic resources, and regional healthcare disparities continue to determine whether precision-guided management can be applied in routine practice.

## 7. Clinical, Economic, and Structural Barriers to Precision-Guided Eradication

### 7.1. Limited Access to Resistance Testing

Although resistance-guided therapy is increasingly recommended by international guidelines, access to AST remains limited in many healthcare systems. Culture-based testing requires endoscopy, specialized microbiology laboratories, trained personnel, and meticulous specimen handling, making routine implementation difficult outside tertiary referral centers [126,127]. Molecular assays provide faster results and simpler workflows but remain unevenly available because of differences in laboratory infrastructure, diagnostic capacity, cost, and reimbursement policies [128,129].

As a result, treatment decisions in many regions continue to rely on empirical regimens guided by regional resistance estimates rather than patient-specific susceptibility data [18]. This gap between guideline recommendations and real-world practice represents one of the principal obstacles to implementing precision-guided *H. pylori* management worldwide.

Furthermore, limited access to susceptibility testing has implications beyond individual patient care. Continued reliance on empirical therapy increases the likelihood of repeated exposure to ineffective antibiotics, potentially contributing to additional resistance selection and reducing future therapeutic options. Expanding access to reliable resistance testing is therefore important for both individualized treatment and antimicrobial stewardship [18,126].

### 7.2. Economic Constraints and Guideline Variability

The implementation of precision-guided management is influenced not only by diagnostic availability but also by economic considerations. Resistance testing increases upfront healthcare costs through laboratory procedures, specialized equipment, and trained personnel. Nonetheless, several economic evaluations suggest that susceptibility-guided treatment may become cost-effective by reducing repeated treatment failures, unnecessary antibiotic exposure, and the need for rescue therapy [130,131]. Importantly, cost-effectiveness is highly context dependent and varies according to regional resistance prevalence, diagnostic costs, healthcare infrastructure, and reimbursement policies.

Implementation is complicated by differences among international guidelines. Although recent consensus reports consistently recognize the value of susceptibility-guided therapy, they differ regarding indications for resistance testing, patient selection, preferred diagnostic methods, and treatment algorithms [3,58]. These differences largely reflect variations in regional AMR patterns, healthcare resources, and access to diagnostic technologies rather than conflicting scientific evidence. Nevertheless, such variability may complicate implementation and create uncertainty for clinicians practicing across different healthcare systems.

The economic value of precision-guided management should also be considered in relation to long-term eradication outcomes. Although susceptibility testing increases the initial cost of care, several studies have shown that tailored therapy may reduce repeated treatment failures, unnecessary antibiotic use, and additional healthcare visits. As a result, individualized treatment can become cost-effective, particularly in regions with high antimicrobial resistance or in patients with previous eradication failure. However, the overall economic benefit varies according to local resistance patterns, testing costs, and available healthcare resources [130,131].

### 7.3. Global Disparities and Future Challenges

Substantial global disparities continue to influence access to contemporary *H. pylori* management. Regions with the highest infection burden frequently have the least access to susceptibility testing, resistance surveillance programs, and newer therapeutic options [18]. Consequently, empirical therapy remains the only practical treatment strategy in many low-resource settings despite increasing AMR.

These inequalities also present important antimicrobial stewardship challenges. Repeated use of empirical regimens in areas where resistance patterns are poorly characterized may accelerate the selection of resistant strains and further reduce future treatment effectiveness [3,18]. Addressing these disparities will require coordinated efforts to strengthen laboratory infrastructure, expand resistance surveillance, improve access to molecular diagnostics, and promote equitable availability of effective eradication therapies.

Future implementation of precision-guided management should therefore focus not only on developing more advanced diagnostic technologies but also on ensuring that these innovations are accessible, affordable, and adaptable across diverse healthcare settings. Without improvements in healthcare infrastructure and international surveillance, the benefits of precision medicine are likely to remain concentrated in well-resourced regions.

The major barriers limiting implementation of precision-guided *H. pylori* eradication strategies and the corresponding opportunities for improvement are summarized in Table 5. Overall, current evidence indicates that the principal barriers to precision-guided eradication are no longer primarily scientific but practical. Although effective diagnostic technologies and individualized treatment strategies are increasingly available, their successful implementation depends on healthcare infrastructure, economic sustainability, laboratory capacity, and coordinated antimicrobial stewardship. Overcoming these challenges will be essential before precision-guided management can become routine clinical practice worldwide.

Addressing these barriers will require not only improvements in healthcare systems but also continued technological innovation. The following section discusses emerging diagnostic and digital technologies that may facilitate broader implementation of precision-guided management.

## 8. Future Directions in Precision *H. pylori* Management

### 8.1. Toward Rapid and Precision-Guided Diagnostics

The transition from empirical treatment toward precision-guided management will depend heavily on the development of diagnostic tools capable of providing rapid, accurate, and clinically actionable information. Although conventional culture-based susceptibility testing remains the reference standard for AMR assessment, its widespread implementation is limited by technical complexity, specialized laboratory requirements, and prolonged turnaround times. These limitations have stimulated growing interest in molecular approaches that can identify both *H. pylori* infection and resistance-associated mutations directly from clinical specimens, thereby facilitating earlier therapeutic decision-making [66,133,134].

Among emerging technologies, PCR-based platforms have demonstrated particular promise for rapid resistance profiling. Early studies showed that real-time PCR can accurately detect key clarithromycin-resistance mutations directly from gastric biopsy samples, providing results within hours rather than days required for conventional culture methods [135,136]. Similarly, fluorescence in situ hybridization and other molecular assays have enabled direct identification of resistant strains in clinical specimens without the need for bacterial isolation [137]. More recently, advances in next-generation sequencing and multiplex molecular testing have expanded the capacity to characterize resistance determinants comprehensively, offering opportunities for broader resistance surveillance and individualized treatment selection [66,138].

An additional area of rapid innovation involves the development of point-of-care diagnostic technologies. Biosensor-based platforms, microfluidic devices, and lab-on-a-chip systems have attracted increasing attention because they combine miniaturization, rapid processing, and reduced dependence on centralized laboratory infrastructure [139,140]. These technologies are designed to simplify diagnostic workflows while maintaining high analytical performance, making them particularly attractive for use in resource-limited settings where access to specialized testing remains restricted. Although these technologies show considerable promise, most remain at different stages of clinical development and require further validation before widespread implementation. Their future clinical value will depend not only on analytical performance but also on affordability, accessibility, regulatory approval, and successful integration into routine healthcare systems [139,140,141]. Recent developments have further incorporated advanced signal amplification strategies and portable detection systems, improving the feasibility of near-patient resistance testing.

Looking ahead, CRISPR-enabled diagnostic platforms represent a particularly promising frontier in *H. pylori* management. These systems offer the potential for highly sensitive and specific detection of pathogen-associated targets while reducing testing complexity and turnaround times. If successfully validated in prospective clinical studies, these platforms could support more individualized treatment decisions by simultaneously confirming infection and detecting clinically relevant resistance markers during the same diagnostic workflow [141]. These advances suggest that future diagnostic strategies will move beyond simple infection detection toward integrated molecular characterization of AMR. Such developments may substantially shorten the interval between diagnosis and treatment selection, providing a critical foundation for the broader implementation of precision-guided *H. pylori* eradication strategies.

### 8.2. AI-Assisted and Data-Driven Therapeutic Selection

The growing availability of clinical, microbiological, and epidemiological data is creating new opportunities for data-driven treatment selection in *H. pylori* management. Traditional therapeutic decisions have largely relied on regional resistance patterns and empirical guideline recommendations. However, advances in AI and machine learning are enabling more individualized approaches that integrate multiple patient- and pathogen-related variables into therapeutic decision-making frameworks [142,143].

Recent developments suggest that AI-assisted clinical decision systems may help identify the most appropriate eradication regimen for individual patients. Using large multinational datasets, machine learning models have demonstrated the ability to incorporate demographic characteristics, treatment history, antibiotic exposure, geographic factors, and resistance-associated variables to generate personalized therapeutic recommendations. These findings illustrate the potential of AI-assisted decision support; however, prospective validation in diverse healthcare settings remains necessary before routine clinical implementation [144].

Beyond regimen selection, predictive analytics may assist in identifying patients at increased risk of treatment failure before therapy is initiated. Machine learning algorithms have shown promising performance in stratifying individuals according to their likelihood of eradication success, thereby facilitating earlier implementation of alternative therapeutic strategies and reducing unnecessary antibiotic exposure [145]. Similar approaches have also been applied to predict infection risk and characterize population-level determinants of disease, illustrating the broader utility of computational models in supporting precision management strategies [146,147].

Future decision-support systems are expected to combine resistance surveillance, molecular diagnostic results, clinical characteristics, and eradication outcomes. Integrating these data into a single platform could support more individualized treatment decisions. Such systems could provide continuously updated therapeutic recommendations that adapt to changing resistance patterns and local epidemiological conditions. Although prospective validation, regulatory oversight, and integration into routine clinical workflows remain necessary, these technologies represent an important step toward more precise, evidence-based, and individualized *H. pylori* management [143,148]. Successful implementation will also depend on standardized data collection, interoperability between diagnostic and clinical information systems, protection of patient privacy, and appropriate regulatory oversight. Addressing these challenges will be essential to ensure that AI-assisted decision support is reliable, transparent, and clinically applicable [143,148].

**Figure 5:**
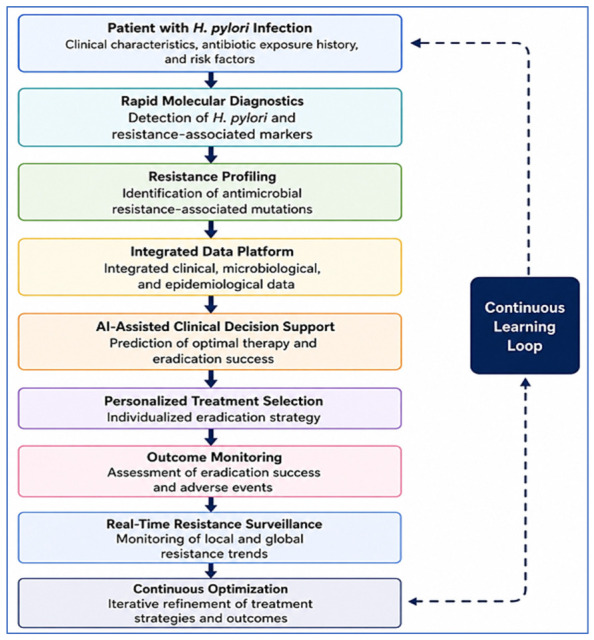
Conceptual framework illustrating the future evolution of precision-guided *H. pylori* management. Rapid molecular diagnostics, AMR profiling, integrated clinical and epidemiological data, AI-assisted decision support, individualized treatment selection, and continuous surveillance interact to support adaptive, evidence-informed management strategies while promoting antimicrobial stewardship.

Ultimately, the future of *H. pylori* management will likely depend on the convergence of rapid molecular diagnostics, real-time resistance surveillance, and AI-assisted clinical decision support. Figure 5 summarizes a conceptual framework illustrating how rapid diagnostics, resistance profiling, integrated data systems, and AI-assisted decision support may converge to enable precision-guided management of *H. pylori* infection. Integrating these technologies may enable more individualized eradication strategies. This approach could improve treatment selection, increase eradication success, and support antimicrobial stewardship.

Overall, future progress in *H. pylori* management will depend not only on technological innovation but also on successful clinical translation. Rapid diagnostics, molecular resistance profiling, AI-assisted decision support, and real-time surveillance have the potential to transform treatment selection. However, their impact will depend on rigorous clinical validation, cost-effectiveness, equitable implementation, and integration into routine healthcare practice. These priorities should guide future research and international collaborative efforts aimed at advancing precision-guided *H. pylori* management.

## 9. Conclusions

*H. pylori* infection remains a major global health challenge, and the continued rise in antimicrobial resistance has reduced the effectiveness of traditional empirical eradication strategies. The available evidence shows that treatment success depends on multiple interacting factors, including antimicrobial resistance, host characteristics, treatment adherence, and regional epidemiology. These findings highlight the need to move beyond a uniform treatment approach toward more individualized management. Precision-guided management provides a practical framework for achieving this goal. Susceptibility testing is an important component, but it should be integrated with previous antibiotic exposure, treatment history, local resistance patterns, patient characteristics, and available healthcare resources to support individualized treatment decisions. Continued advances in molecular diagnostics, resistance surveillance, and treatment optimization are expected to further improve eradication success and strengthen antimicrobial stewardship. *H. pylori* management is no longer simply a matter of developing new eradication regimens. Greater progress is likely to come from selecting existing therapies more appropriately for each patient. Greater progress is likely to come from selecting existing therapies more appropriately for each patient. This requires combining resistance testing, clinical characteristics, previous treatment history, and local resistance patterns whenever possible. From a practical perspective, eradication therapy should be individualized whenever reliable clinical or resistance information is available. Where susceptibility testing is not feasible, treatment should follow current regional guideline recommendations and local resistance data. This balanced approach allows clinicians to optimize treatment while adapting to the resources and resistance patterns of their local healthcare setting. In our view, the future of *H. pylori* management depends less on developing entirely new treatment regimens than on making better use of the tools already available. Integrating accurate diagnostics, evidence-based treatment selection, and responsible antimicrobial stewardship offers the most realistic strategy for improving eradication success and preserving the effectiveness of existing antibiotics. Future research should focus on making these precision-guided approaches more accessible, affordable, and applicable across diverse healthcare settings.

## Figures and Tables

**Table 1:** Representative AMR patterns and therapeutic implications across geographic regions and clinical populations.

Region/Population	Clarithromycin Resistance	Metronidazole Resistance	Fluoroquinolone Resistance	Multidrug Resistance	Therapeutic Implications	Refs.
WHO regions (global meta-analysis)	Primary resistance exceeds the 15% threshold in most WHO regions, with the highest prevalence in the Eastern Mediterranean region	Approximately 56% in the Eastern Mediterranean region	Exceeds 15% in several regions	Reported worldwide	Consider alternatives to empirical clarithromycin-based triple therapy. Base treatment selection on local resistance data whenever available.	[17]
Europe	Marked variation between countries	Common in several countries	Progressive increase reported	Present in multiple populations	Adapt first-line therapy to regional resistance patterns and consider susceptibility-guided treatment when feasible.	[18]
Asia–Pacific region	Frequently exceeds the accepted therapeutic threshold	High prevalence in several countries	High prevalence in multiple populations	Dual resistance frequently reported	Prefer BQT or susceptibility-guided treatment in regions with a high resistance burden.	[21]
China (Shandong Province)	A2143G mutation frequently detected	rdxA/frxA alterations commonly identified	gyrA mutations commonly reported	Dual and triple resistance identified	Use molecular resistance testing whenever available to improve treatment selection and reduce reliance on empirical rescue therapy.	[22]
Middle Eastern populations	Frequently exceeds 15%	High prevalence reported	Elevated prevalence observed	Reported in several studies	Avoid empirical clarithromycin-based triple therapy where resistance is high and consider alternative first-line regimens.	[25]
African populations	Limited surveillance data available	Frequently elevated	Regional data remain insufficient	Poorly characterized	Expand regional surveillance to support evidence-based treatment recommendations.	[27]
Pediatric populations	Rising prevalence observed	Frequently elevated	Variable prevalence	Emerging concern	Monitor resistance trends closely, as increasing resistance may further restrict future treatment options.	[24]
International clinical cohorts	Associated with lower eradication rates	Contributes to treatment failure	Associated with rescue therapy failure	Increasingly recognized	Incorporate regional resistance data into treatment selection to improve eradication success and support individualized management.	[26]

**Table 2:** Major host-, lifestyle-, bacterial-, and treatment-related determinants contributing to *H. pylori* eradication failure and their clinical implications.

Domain	Determinant	Clinical Impact	Refs.
Host-related	Poor adherence	Frequently the strongest modifiable predictor of treatment failure and reduced eradication success.	[31,32,33]
	Treatment intolerance	Reduces treatment adherence and completion, thereby lowering eradication success.	[34,36]
	Variable acid suppression	Limits antibiotic activity by reducing intragastric pH control and decreases treatment efficacy.	[37,38]
	CYP2C19 polymorphisms	Influence proton pump inhibitor metabolism, resulting in variable treatment response.	[37,38]
Lifestyle/Metabolic	Obesity and metabolic dysfunction	Associated with lower eradication rates, possibly because of altered drug pharmacokinetics, chronic inflammation, and metabolic impairment.	[39,41]
Bacterial	Biofilm formation	Reduces antibiotic penetration and promotes bacterial persistence despite treatment.	[8,46]
	Heteroresistance	May result in incomplete eradication because susceptible and resistant bacterial subpopulations coexist.	[8]
	Intracellular persistence	Protects bacteria from antimicrobial exposure and host immune responses.	[48,49]
	High bacterial burden	Increases the likelihood of persistent infection and lowers eradication success.	[51,52]
Treatment-related	Inadequate treatment duration	Reduces eradication efficacy by providing insufficient antimicrobial exposure.	[53,54]
	Suboptimal dosing	Limits bacterial clearance and increases the risk of persistent infection.	[53]
	Complex multidrug regimens	Increase treatment burden, reduce adherence, and compromise treatment completion.	[54,56]

**Table 3:** Comparison of phenotypic and molecular resistance-testing approaches in *H. pylori* management.

Characteristic	Phenotypic Susceptibility Testing	Molecular Resistance Testing	Key Clinical Implication	Refs.
Principle	Direct measurement of bacterial growth in the presence of antibiotics	Detection of resistance-associated genetic mutations	Phenotypic testing measures expressed resistance, whereas molecular testing predicts resistance from validated genetic markers.	[60,65]
Sample requirement	Viable *H. pylori* isolate obtained from gastric biopsy culture	Gastric biopsy DNA or cultured isolates	Molecular testing can be performed without successful bacterial culture, increasing its clinical applicability.	[60,66]
Main methods	Agar dilution, E-test	PCR, real-time PCR, line probe assays, targeted sequencing, whole-genome sequencing	Method selection depends on laboratory expertise, available resources, and the clinical question.	[62,64,69]
Resistance information provided	Susceptibility profile for tested antibiotics	Identification of specific resistance-associated mutations	Phenotypic testing provides broader susceptibility data, whereas molecular testing is most effective for targeted resistance detection.	[67,68]
Turnaround time	Longer because bacterial culture is required	Rapid, often within hours to a few days	Rapid molecular testing may support earlier treatment optimization.	[61,65]
Clarithromycin-resistance detection	Direct phenotypic confirmation	High diagnostic accuracy for 23S rRNA mutations	The most established clinical application of molecular resistance testing.	[67,68]
Fluoroquinolone resistance detection	Direct phenotypic confirmation	Detection of gyrA mutations associated with resistance	Supports selection of appropriate salvage therapy when fluoroquinolone resistance is suspected.	[67,68]
Detection of novel or uncommon resistance mechanisms	Possible when resistance is expressed phenotypically	Limited to known genetic targets	Phenotypic testing remains important when resistance mechanisms are uncertain or previously unrecognized.	[60,69]
Genotype–phenotype concordance	Reflects observed antimicrobial susceptibility	Genetic mutations may not always predict expressed resistance	Discordance is most relevant for antibiotics such as metronidazole, where multiple resistance mechanisms exist.	[68,69]
Technical requirements	Specialized culture facilities and trained personnel	Molecular diagnostic platform and technical expertise	Resource availability, laboratory infrastructure, and local expertise influence test selection.	[61,70]
Current clinical role	Reference standard for comprehensive susceptibility assessment	Increasingly used to support individualized treatment selection	Both approaches are complementary and should be selected according to the clinical setting and available resources.	[12,75]

**Table 4:** Comparative summary of contemporary first-line, rescue, and optimization strategies for *H. pylori* eradication.

Regimen	Clinical Role	Key Advantages	Key Limitations	Preferred Clinical Setting	Refs.
BQT	First-line and rescue therapy	High eradication efficacy; activity maintained despite clarithromycin resistance; extensive clinical experience	High pill burden; treatment complexity may reduce adherence	Preferred empirical regimen in regions with high clarithromycin resistance, previous macrolide exposure, or when susceptibility testing is unavailable.	[3,58]
Vonoprazan-based therapy	Optimized first-line therapy	Rapid and sustained acid suppression; simplified administration; high eradication efficacy	Limited availability; higher cost in some healthcare systems	Prefer when vonoprazan is available, particularly in patients who may benefit from enhanced acid suppression or simplified treatment.	[85,88]
High-dose dual therapy (HDDT)	Simplified first-line alternative	Reduced antibiotic exposure; favorable safety profile; simplified antibiotic regimen	Requires strict dosing adherence and effective acid suppression	Consider when minimizing unnecessary antibiotic exposure is a priority and the patient is likely to adhere to frequent dosing.	[93]
Levofloxacin-based therapy	Second-line salvage therapy	Well-established rescue option; generally well tolerated	Declining efficacy in regions with high fluoroquinolone resistance	Reserve for patients with susceptible strains or in regions with low fluoroquinolone resistance, preferably after susceptibility testing.	[99]
Rifabutin-based therapy	Later-line rescue therapy	Active against many multidrug-resistant strains; useful after multiple treatment failures	Potential hematologic toxicity; antimicrobial stewardship concerns	Reserve for carefully selected patients with refractory infection after failure of standard rescue regimens.	[112]
Adjunctive probiotic supplementation	Supportive treatment optimization	Improves treatment tolerability; may reduce gastrointestinal adverse events; modest improvement in eradication rates	Benefits vary according to probiotic strain, dose, and treatment protocol	Use as adjunctive therapy in patients at increased risk of treatment-related adverse events or poor treatment adherence.	[117,120]

**Table 5:** Major barriers limiting implementation of precision-guided *H. pylori* eradication strategies and recommended approaches to overcome them.

Barrier	Clinical Impact	Recommended Strategy	Refs.
Limited access to AST	Sustains reliance on empirical therapy and reduces treatment individualization	Expand access to both culture-based and molecular susceptibility testing.	[126,127]
Laboratory and infrastructure limitations	Restrict diagnostic capacity and delay implementation of precision-guided management	Strengthen laboratory networks, regional reference centers, and diagnostic infrastructure.	[129,132]
Delayed availability of resistance results	Limits timely optimization of therapy	Implement rapid molecular diagnostic platforms and shorter reporting pathways.	[128,129]
Variation among international guidelines	Produces differences in testing indications and treatment algorithms	Harmonize recommendations while maintaining regional flexibility based on local resistance epidemiology.	[3,58]
Inadequate resistance surveillance	Weakens evidence-based empirical therapy and antimicrobial stewardship	Expand national and international surveillance networks with standardized reporting.	[18,126]
Economic and reimbursement constraints	Limit routine adoption of susceptibility-guided therapy	Develop reimbursement policies and prioritize cost-effective targeted testing.	[130]
Global inequities in healthcare resources	Restrict access to diagnostics and optimized therapies	Improve equitable access to diagnostic technologies, effective treatments, and laboratory capacity.	[3,18]

## Data Availability

No new data were created or analyzed in this study.
